# The paracrine induction of prostate cancer progression by caveolin-1

**DOI:** 10.1038/s41419-019-2066-3

**Published:** 2019-11-04

**Authors:** Chun-Jung Lin, Eun-Jin Yun, U-Ging Lo, Yu-Ling Tai, Su Deng, Elizabeth Hernandez, Andrew Dang, Yu-An Chen, Debabrata Saha, Ping Mu, Ho Lin, Tsai-Kun Li, Tang-Long Shen, Chih-Ho Lai, Jer-Tsong Hsieh

**Affiliations:** 1 Department of Urology, University of Texas Southwestern Medical Center, Dallas, TX 75390 USA; 2 Division of Integrative Bioscience and Biotechnology, POSTECH, Pohang, 37673 Republic of Korea; 3 Department of Plant Pathology and Microbiology, National Taiwan University, Taipei, Taiwan; 4 Department of Molecular Biology, University of Texas Southwestern Medical Center, Dallas, TX 75390 USA; 5 Department of Radiation Oncology, University of Texas Southwestern Medical Center, Dallas, TX 75390 USA; 6 Department of Life Sciences, National Chung Hsing University, Taichung, Taiwan; 7 Department and Graduate Institute of Microbiology, National Taiwan University, Taipei, Taiwan; 8 Department of Microbiology and Immunology, Graduate Institute of Biomedical Sciences, College of Medicine, Chang Gung University, Taoyuan, Taiwan; 9 Department of Biotechnology, Kaohsiung Medical University, Kaohsiung, Taiwan

**Keywords:** Cancer stem cells, Prostate cancer

## Abstract

A subpopulation of cancer stem cells (CSCs) plays a critical role of cancer progression, recurrence, and therapeutic resistance. Many studies have indicated that castration-resistant prostate cancer (CRPC) is associated with stem cell phenotypes, which could further promote neuroendocrine transdifferentiation. Although only a small subset of genetically pre-programmed cells in each organ has stem cell capability, CSCs appear to be inducible among a heterogeneous cancer cell population. However, the inductive mechanism(s) leading to the emergence of these CSCs are not fully understood in CRPC. Tumor cells actively produce, release, and utilize exosomes to promote cancer development and metastasis, cancer immune evasion as well as chemotherapeutic resistance; the impact of tumor-derived exosomes (TDE) and its cargo on prostate cancer (PCa) development is still unclear. In this study, we demonstrate that the presence of Cav-1 in TDE acts as a potent driver to induce CSC phenotypes and epithelial–mesenchymal transition in PCa undergoing neuroendocrine differentiation through NFκB signaling pathway. Furthermore, Cav-1 in mCRPC-derived exosomes is capable of inducing radio- and chemo-resistance in recipient cells. Collectively, these data support Cav-1 as a critical driver for mCRPC progression.

## Introduction

Prostate cancer (PCa) is the most commonly diagnosed cancer and the second leading cause of death in the United States^1^. Clinical treatment for primary PCa includes radical prostatectomy, hormonal therapy, and radiation. For metastatic PCa into bones^2,3^, androgen-deprivation therapy is a standard of care. However, most prostate tumors eventually relapse to an end-stage castration-resistant prostate cancer (CRPC). Despite of second line of androgen-deprivation therapy or chemotherapy, the life expectancy is ~ 3–5 years and eventually leads to death. Critically, the cell of origin and molecular drivers of CRPC are not fully characterized, which highlight an urgent clinical need to develop mechanism-based therapies to improve overall survival of CRPC patients.

Exosomes are nano-scale extracellular vesicles derived from the endosomal system^4^. Exosomes are formed by the inward budding of multivesicular bodies further released from the cell, and following the fusion of multivesicular bodies with recipient cell plasma membrane^5^. Therefore, the exosome acts as a delivery vehicle that can carry different signaling molecules in a paracrine or endocrine manner. A recent study has shown that tumor-derived exosomes (TDE) containing integrins are the key drivers for determining organotropic metastasis^4^. Overall, TDE have been shown to play a critical role in promoting cancer progression and drug resistance^6,7^. For example, mesenchymal stem cell-derived exosomes can activate quiescent dormant breast cancer in bone marrow or drug resistance of cancer^8,9^. Also, our recent study has shown that the delivery of a specific protein factor by TDE into PCa cells can induce neuroendocrine differentiation (NED) of recipient cells^10^, suggesting a role of TDE in PCa progression.

Cancer stem cells (CSC) have an impaired homeostatic control that leads to malignant phenotypes such as immortalization, de-differentiation and multipotency, uncontrolled growth, and anti-apoptosis^11,12^, which in turn are associated with cancer metastasis owing to epithelial–mesenchymal transition (EMT) or cancer recurrence owing to treatment resistance^13^. Many studies indicate that CRPC exhibits many similar phenotypes of CSC^14,15^, suggesting that PCa may be induced to undergo de-differentiation during androgen-deprivation therapy. Previously, we observed that elevated caveolin-1 (Cav-1) can promote PCa growth in a paracrine manner^16^, indicating that Cav-1 can function on neighboring cell via secretion. This study, for the first time, demonstrates that the TDE can deliver Cav-1 into recipient cells in a paracrine fashion.

Caveolin protein family, including caveolin-1, -2, and -3, is the major component of caveolae^17,18^. Among them, Cav-1 has been extensively characterized and is known to participate in multiple cellular processes, including cell cycle regulation, signal transduction, endocytosis, and cholesterol trafficking/efflux^18^. In PCa, elevated Cav-1 is correlated with PCa progression^19^. In addition, recent studies have shown that Cav-1 expression leads to chemotherapy resistance in multiple cancers^20,21^, and has even been indicated to promote CSC properties in lung cancer^22,23^. Our data indicate that increased Cav-1 in PCa cells by either gene transfection or TDE delivery can increase CSC phenotypes in vitro and along with elevated cancer-initiating activity in vivo. Furthermore, we delineate the mechanism of action of Cav-1 in promoting EMT and NED phenotype mediated through NFκB signaling pathway.

## Results

### Caveolin-1 (Cav-1) promotes CSC phenotypes

**Fig. 1:**
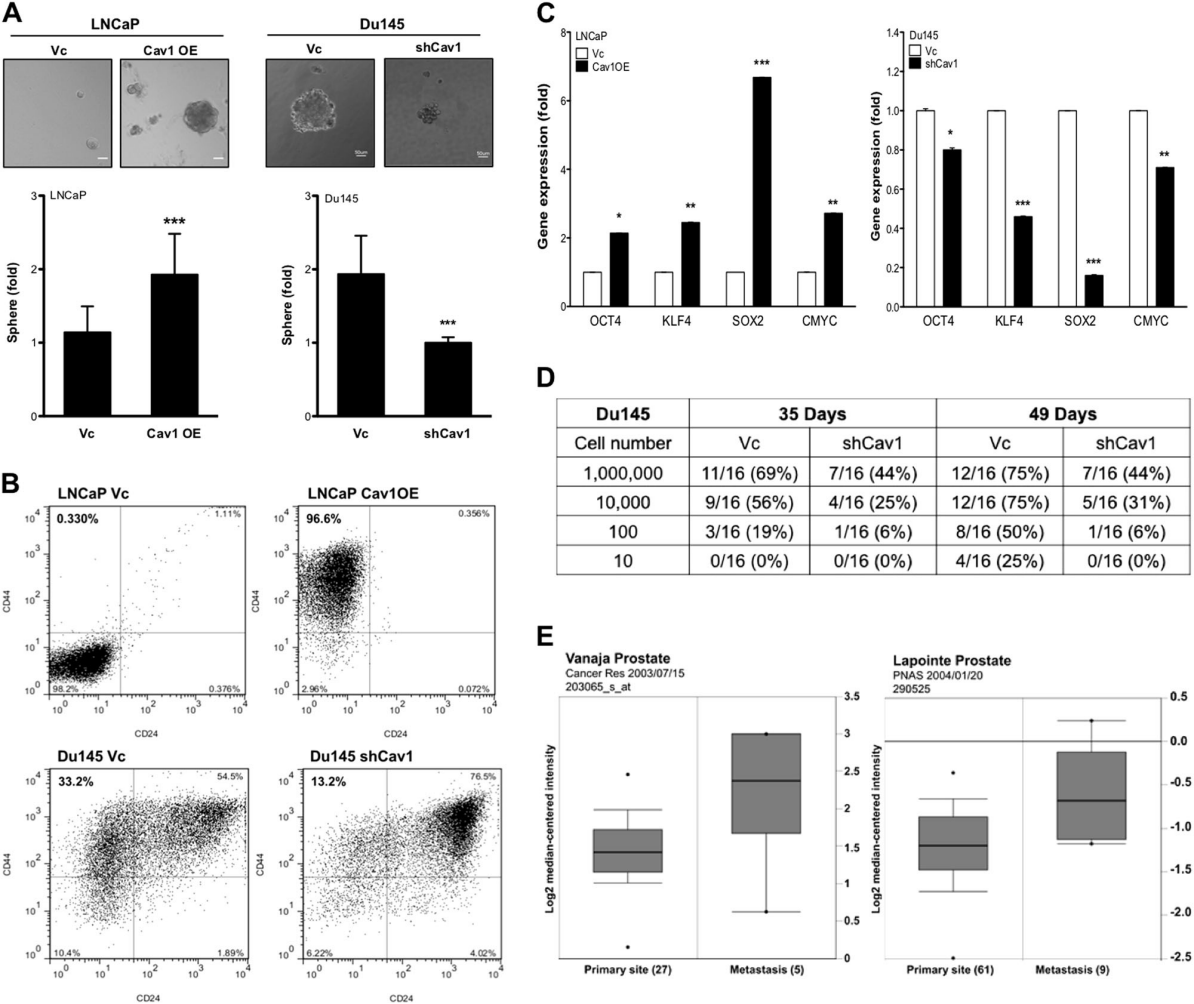
Cav-1 increases CSC subpopulation. **a** Prostate sphere formation of LNCaP (Vc vs. CavOE) and Du145 (Vc vs. shCav1) was determined after 14 days cultured in ultralow attachment plate. Number of spheres was counted and normalized with control cells (e.g., LNCaP Vc or Du145 shCav1), respectively. Data were shown as mean ± SD (*n* > 10), ***P < 0.001. Scale bar = 50 μm. **b** CSC subpopulation was determined by Flow cytometry based on CD24^low^ and CD44^high^ population. **c** The gene expression of Yamanaka factors was measured by qRT-PCR. Data were shown as mean ± SD (*n* > 10), *P < 0.5, **P < 0.01, ***P < 0.001. **e** In vivo tumorigenicity of Du145 Vc vs. shCav1 cells was measured at Day 35 and Day 49 post injection. **d** A serial dilution of Du145 Vc or shCav1 cells was subcutaneously injected into immune-deficient mice. Tumor incidence was analyzed after 35 days and 49 days of injection. **e** Clinical correlation of Cav-1 mRNA expression in PCa patients from two cohorts was obtained by using Oncomine Database. Cav-1 mRNA expression was compared between primary site and metastasis site of tumor

Elevated Cav-1 levels are detected in tumor tissues and serum from PCa patient and correlate with disease progression^19,24^. Our previous study also demonstrates that Cav-1 can promote in vivo tumor growth of PCa in an endocrine manner^16^. Also, we observed a significantly elevated Cav-1 expression in several CRPC cell lines (PC3, Du145, and 22Rv1), when compared with androgen-responsive PCa cell lines (LAPC4, VCaP, and LNCaP) (Supplemental S1A). Thus, we hypothesized that Cav-1 can promote the recurrent CRPC by inducing stemness phenotypes. Indeed, by ectopically expressing Cav-1 in LNCaP (Cav1OE) (Supplemental S1B), both the number and the size of prostate spheres significantly increased compared with the vector control (Vc) (Fig. 1a). In contrast, by knocking down Cav-1 in Du145 (shCav1) (Supplemental S1C), both the number and the size of prostate spheres significantly decreased compared with control (Fig. 1a). The subpopulation of CD44^+^/CD24^−^ cells represent CSC in PCa^14,25^; we found an increase in this subpopulation in LNCaP Cav1OE (96.6%) compared with LNCaP Vc (0.3%) (Fig. 1b). In addition, we observed that this subpopulation was decreased in Du145 shCav1 cells (13.2%) compared with Du145 Vc cells (33.2%) (Fig. 1b). We also confirmed the expression of CD24 and CD44 mRNA in these cell models; the results were consistent with their protein levels (Supplemental S1D), suggesting that Cav-1 signaling pathway can mediate CD24 and CD44 gene expression. Furthermore, we also found the elevated expression of Yamanaka factors (OCT4, KLF4, SOX2, and C-MYC), key drivers of pluripotent stem cell in both LNCaP Cav1OE and Du145 compared with each control (Fig. 1c). In addition, aldehyde dehydrogenase 1A1 (ALDH1A1) has been used as the CSC marker in PCa^26,27^. Consistently, the expression of ALDH1A1 is significantly higher in Cav-1^high^ cells (LNCaP Cav1OE, Du145 Vc) cell compared with their counterpart (Supplemental S1E). To further compare the in vivo cancer-initiating capability of Cav-1^high^ (Vc) vs. Cav-1^low^ (shCav1) in Du145, we injected a very low cell number into immune-deficient mice and found that Cav-1^low^ cells were less tumorigenic compared with Cav-1^high^ cells at low cell number (Fig. 1d). Despite of different tumor incidence, the tumor volume is not significantly different between these two cells at Week 5 (Du145 Vc = 522 ± 432 mm^3^; Du145 shCav1 = 51.3 ± 33.2 mm^3^; *p* = 0.173) (Fig. 1d). Clinically, data from two cohorts of PCa obtained from Oncomine database (Vanaja Prostate dataset [203065_s_at]^28^ and Lapointe Prostate dataset [290525]^29^) indicated that Cav-1 mRNA expression was significantly higher in metastasis specimens compared with the primary tumor specimens (Fig. 1e). Overall, these data support the notion that Cav-1 is able to promote CSC phenotypes.

### Cav-1 is a key mediator for NED in PCa with Rb and TP53 mutation

**Fig. 2:**
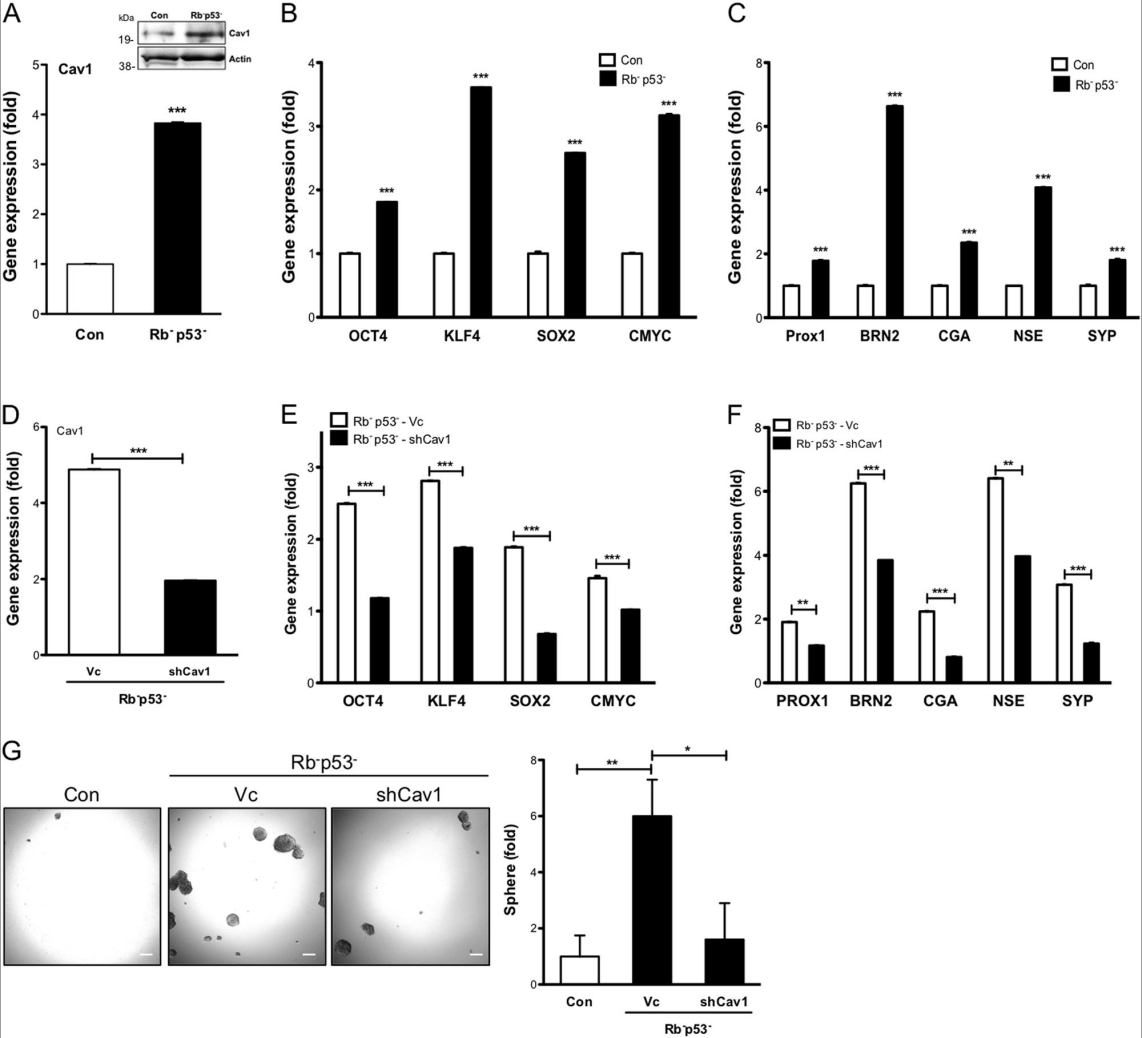
Induction of Cav-1 in PCa carrying p53 and Rb mutation correlates with CSC and NED-related gene expression. The expression profile of **a** Cav-1 in mRNA and protein expression level, **b** Yamanaka factors (OCT4, KLF4, SOX2, CMYC), and **c** NED-related genes (PROX1, BRN2, CGA, NSE, SYP) were determined in LNCaP Con vs. LNCAP (p53^−^, Rb^−^) by qRT-PCR. The gene expression profile of **d** Cav-1, **e** Yamanaka factors genes (OCT4, KLF4, SOX2, CMYC), and **f** NED-related genes (PROX1, BRN2, CGA, NSE, SYP) were determined by qRT-PCR in LNCaP (p53^−^, Rb^−^) cells after Cav-1 knockdown using specific shRNA. **g** The sphere formation in LNCaP Con, LNCAP (p53^−^, Rb^−^)-Vc and LNCAP (p53^−^, Rb^−^)-shCav1 cells. Scale bar = 100 μm. All data were shown as mean ± SD (*n* > 10), *P < 0.5, **P < 0.01, ***P < 0.001

Genetic alterations in both Rb and TP53 genetic alterations are commonly found in CRPC patients^30^. Experimentally, both Rb1 and TP53 double knockdown (shRb1/TP53) in LNCaP cells acquire anti-androgen resistance and lineage plasticity evidenced by elevated Sox2 expression^31^. Also, Rb/TP53 double knockdown mouse prostate cells can grow organoids in the three-dimensional culture system, which suggest their stem cell behavior^32,33^. As SOX2 is one of the Yamanaka factors, we decided to examine whether Cav-1 is involved in regulating the lineage plasticity driven by Rb1- and TP53-deficient in CRPC. Indeed, the result indicated a significant elevation of Cav-1 in Rb^−^p53^−^ cells (Fig. 2a). Interestingly, p53 KD alone is sufficient to increase the expression of Cav-1, whereas Rb1 KD alone only has a minimal effect. In addition, p53 or Rb1 KD contributes equally to the expression of OCT4, SOX2, whereas Rb1 KD is more potent in increasing both CMYC and KLF4 expression (Supplemental S2A, B). These data suggest that loss of both Rb1 and p53 are required for CSC phenotype in PCa. Similarly, the expressions of Yamanaka factors and ALDH1A1 were elevated in Rb^−^p53^−^ cell compared with Vc cell (Fig. 2b, Supplemental S2C). In addition, expression of the neuroendocrine driver genes (PROX1, BRN2)^34,35^ and biomarkers (CGA, NSE, and SYP)^36–38^ were increased in Rb^−^p53^−^ cell compared with Vc cell (Fig. 2c). To further elucidate the central role of Cav-1 contributing to the elevation of stem cell and NE factors, Cav-1 was knocked down in the Rb^−^p53^−^ cells (shCav1) (Fig. 2d) and caused significantly decreased expression of Yamanaka genes (Fig. 2e) and NED-related genes (Fig. 2f). Specifically, we examined prostate sphere formation in these cells and double knockdown of Rb and p53 significantly increase sphere formation, whereas knockdown Cav-1 in Rb^−^p53^−^ cells significantly reduced prostate sphere formation (Fig. 2g). The induction of NED-related gene expression was observed in LNCaP (Cav1OE); in contrast, the reduction of these genes was observed in Du145 (shCav1) cells (Supplemental S2D).

### Exogenous Cav-1 protein promotes prostate sphere formation

**Fig. 3:**
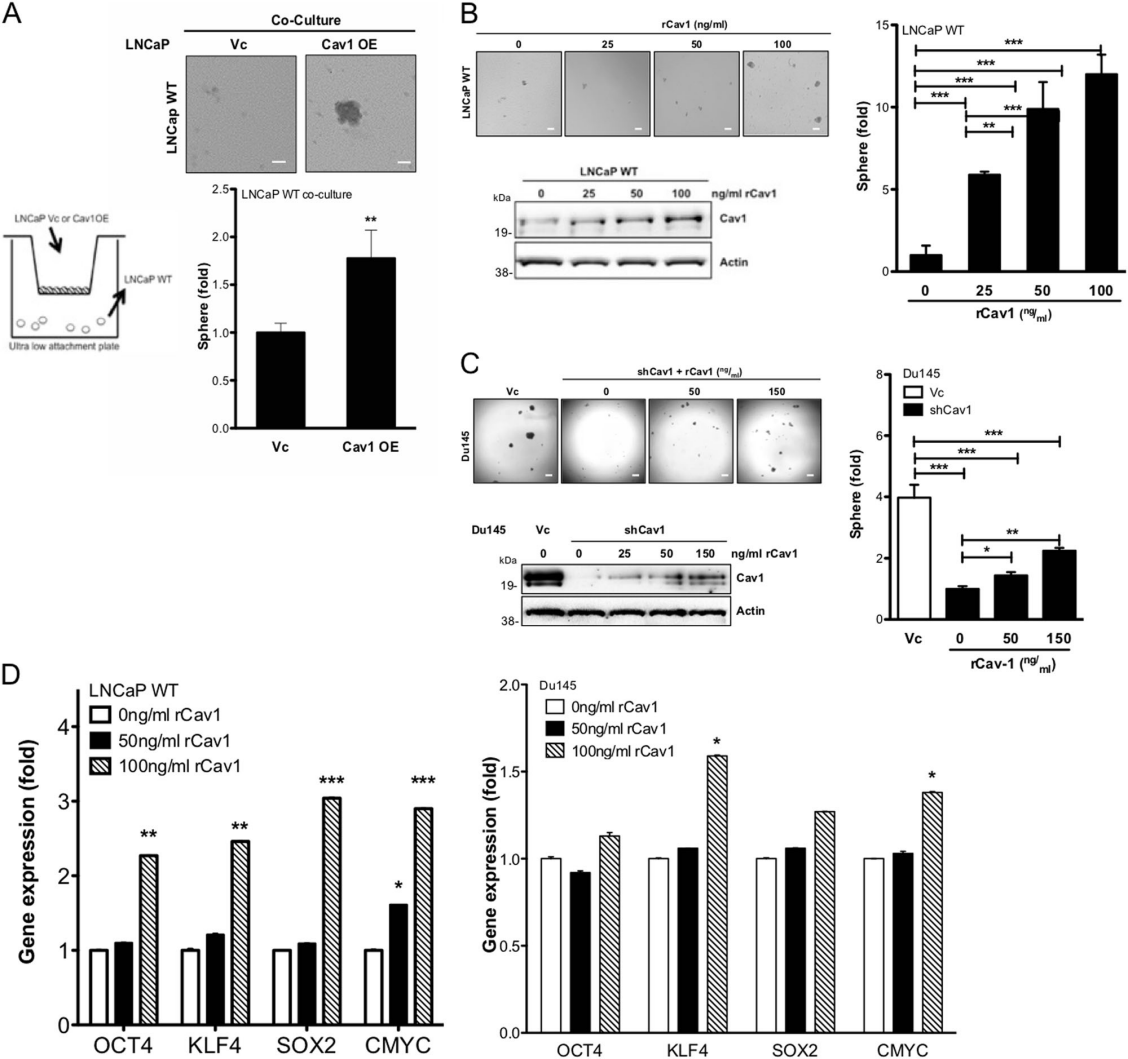
Exogenous rCav-1 promotes CSC phenotypes. **a** Prostate sphere formation of WT LNCaP under the influence of Cav-1-expressing cells was determined after plating LNCaP WT (500 cells) onto the bottom of an ultralow attachment 24-well plate and either LNCaP Vc or Cav1OE (5 × 10^4^ cells) onto a Transwell (3.0-µm polycarbonate). Number of spheres was determined 14 days after co-culture. Scale bar = 50 μm. **b** Upper panel: prostate sphere formation of LNCaP WT incubated with rCav-1 (from 0 to 100 ng/ml) was determined after 14 days. Lower panel: rCav-1 uptake by WT LNCaP was analyzed by western blot. Scale bar = 100 μm. **c** Upper panel: prostate sphere formation of Du145 shCav1 (low Cav-1 expression) incubated with rCav-1 (from 0 to 150 ng/ml) was determined after 14 days. Lower panel: rCav-1 uptake by Du145 shCav1was analyzed by western blot. Scale bar = 100 μm. **d** The gene expression profile of Yamanaka factors gene (Left panel) and NED-related genes (Right panel) was examined 48h post treatment. Scale bar = 50 μm. All data were shown as mean ± SD (*n* > 10), *P < 0.5, **P < 0.01, ***P < 0.001

Although Cav-1 is associated with the cell membrane, some evidence indicates intact Cav-1 can be secreted into the extracellular milieu^16,39^. Therefore, we examined the functionality of secretory Cav-1 in inducing CSC phenotype. We plated either LNCaP Vc or Cav1OE cells on the upper chamber of Transwell and wild type (WT) LNCaP cells on the bottom chamber; a significant higher number of spheres was found in Cav1OE co-culture condition (Fig. 3a). In addition, human recombinant Cav-1 (rCav-1, 25–100 ng/ml) was able to increase prostate sphere formation of WT LNCaP cells in a dosage-dependent manner, which was confirmed by the presence of rCav-1 protein in LNCaP cells (Fig. 3b). Similarly, rCav-1 could partially restore prostate sphere formation of Du145 shCav1 cells, which was correlated with slightly increased Cav-1 levels in Du145 shCav1 cells (Fig. 3c). Consistent with prostate sphere formation, the incremental rCav-1 could induce the expression of Yamanaka factors in WT LNCAP cells or Du145 shCav1 (Fig. 3d). Taken together, our data indicate that soluble Cav-1 can induce the CSC phenotype in a paracrine manner.

### TDE-containing Cav-1 promotes CSC phenotypes

**Fig. 4:**
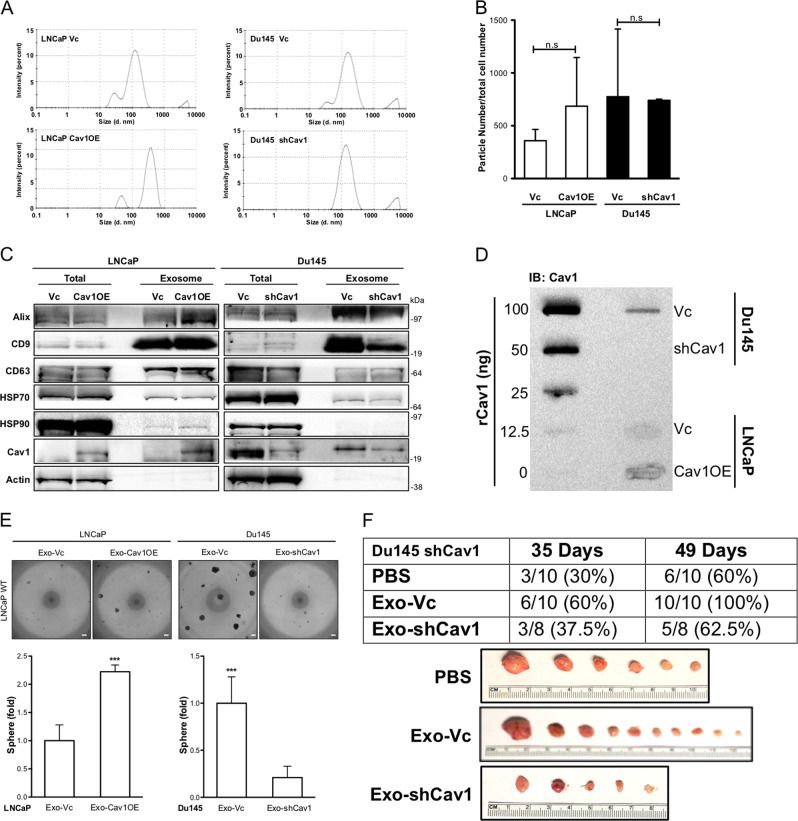
Cav-1 delivered by exosomes promotes CSC phenotypes. Characterization of exosomes purified from LNCaP (Vc or Cav1OE) or Du145 (Vc, shCav1) based on **a** particle size, **b** particle number per cell, and **c** exosome markers (Alix, CD9, CD63, HSP70, HSP90). **d** The amount of Cav-1 in purified exosomes was determined by dot blot assay. Cav-1 concentration was analyzed by rCav-1 (0–100 ng) standard curve. **e** Prostate sphere formation of WT LNCaP was determined after treating with purified exosomes from LNCaP (Vc or Cav1OE) or Du145 (Vc, shCav1) for 14 days of treatment. Scale bar = 100 μm. Data were shown as mean ± SD (*n* > 10), n.s., non-significant, *P < 0.5, **P < 0.01, ***P < 0.001. **f** Du145 shCav1 cells were subcutaneously injected in immune-deficient mice then treated with PBS or TDEs purified from Du145 Vc (Exo-Vc) or Du145 shCav1 (Exo-shCav1), twice-a-week and tumor incidence was recorded at Week 5 and Week 7 post injection. And all tumors were collected at Week 7

Knowing that exogenous Cav-1 protein can induce prostate sphere formation in a paracrine manner, we hypothesize that Cav-1 can be delivered in an endocrine manner via TDE. We purified exosome from conditioned medium (CM) collected from Cav-1-positive or -negative cells and found the similar exosome size (LNCaP Vc: 167.8 ± 14.4 nm, LNCaP Cav1OE: 172.9 ± 12.5 nm; Du145 Vc: 142.9 ± 4.5, Du145 shCav1: 147.6 ± 3.4) among these cells (Fig. 4a). Also, no significant difference of TDE number produced by LNCaP Cav1OE (358.27 ± 106.40 particles/cell) vs. LNCaP Vc (685.23 ± 460.59 particles/cell), or in Du145 Vc (775.42 ± 640.77 particles/cell) vs. shCav1 (741.15 ± 10.39 particles/cell) (Fig. 4b), suggesting that the presence of Cav-1 may not alter TDE biogenesis. Based on the reported markers of TDE including Alix, CD9, CD63, HSP70 and HSP90^40,41^, only Alix and CD9 detected in PCa TDE suggested that they are associated with PCa TDEs as potential biomarkers (Fig. 4c). Indeed, the presence of Cav-1 was detected in PCa TDEs; we further quantified the amount of Cav-1 in TDE; an ~ 20–30 ng Cav-1 protein was detected from TDE of LNCaP or Du145 respectively (Fig. 4d) based on the standard curve (Supplemental S3A). To examine the activity of Cav-1 from PCa TDE, 100 μg of TDE containing the same amount of Cav-1 as the previous experiments using rCav-1 were added into WT LNCaP as a recipient cell and the data demonstrated that TDE derived from Cav-1-positive cells could significantly induce prostate sphere formation (Fig. 4e). Consistently, based on TDE markers, we confirmed the presence of Cav-1 only in the exosome fraction but not in exosome depleted fraction [Exo(-)CM] (Supplemental S3B); the Exo(-)CM was not able to induce prostate sphere formation (Supplemental S3C). Furthermore, the in vivo data demonstrated that TDE-containing Cav-1 (Exo-Vc) could significantly increase the tumor incidence of Du145 shCav1 cells compared with phosphate-buffered saline (PBS) or TDE purified from Du145 shCav1 (Exo-shCav1). (Fig. 4f). Taken together, our data indicate that TDE can deliver Cav-1 into PC cells to elicit CSC phenotypes in recipient cells.

### Cav-1 elicits NFκB cascade leading to CSC and NED phenotypes

**Fig. 5:**
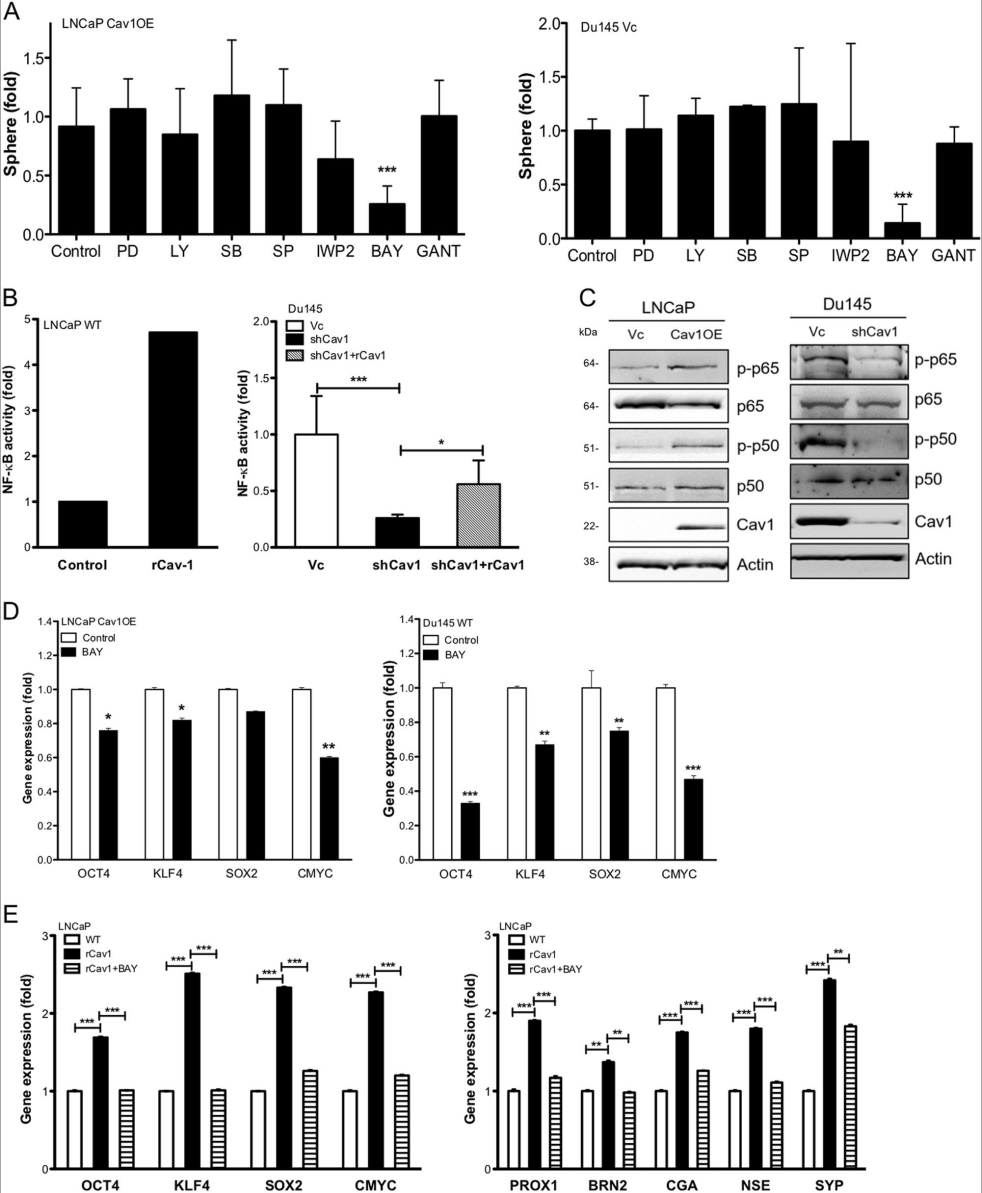
Cav-1 promotes CSC phenotypes through NFκB signaling pathway. **a** The effect of small molecule inhibitors (PI3K/Akt: LY294002 (1 µm); Erk: PD98059 (2 µm), p38: SB202190 (100 nm); JNK: SP600125 (100 nm); Wnt: IWP2 (30 nm); NFκB: BAY11-7082 (5 µm); Gli1/2: GANT (5 µm) on prostate sphere formation of LNCaP Cav1OE and Du145 Vc cells. **b** The effect of rCav-1 on NFκB transcriptional activities was determined after transfecting luciferase reporter gene construct into WT LNCaP or Du145 (Vc or shCav1) cells. Relative luciferase activities were measured and then normalized with Renilla luciferase activities in each sample. **c** The activation status of NFκB pathway was determined by western blot. **d** The effect of NFκB inhibitor (BAY, 5 µm) on gene expression of Yamanaka factors in LNCaP Cav1OE or Du145 Vc cell was determined by qRT-PCR 24h after treatment. **e** The effect of NFκB inhibitor (BAY, 5 µm) on the expression of Yamanaka factors or NED-related genes in WT LNCaP cells treated with rCav-1 was determined by qRT-PCR. Data were shown as mean ± SD (*n* > 10), *P < 0.5, **P < 0.01, ***P < 0.001

Cav-1 is known to be able to activate multiple signaling pathways^42^. To elucidate the signaling pathway leading to CSC or NED phenotypes, we explored a panel of inhibitors of specific signaling pathway such as PI3K/Akt (LY294002), Erk (PD98059), p38 (SB202190), JNK (SP600125), Wnt (IWP2), NFκB (BAY11-7082), and Gli1/2 (GANT) with the optimal concentration that can effectively inhibit each target (Supplemental S4A) but does not alter cell growth (Supplemental S4B). NFκΒ inhibitor demonstrated the most significant inhibitory activity in sphere formation (Fig. 5a). Similar results were also found by using specific siRNA to p50 or p65; knockdown of p65 or p50 significantly decreased prostate sphere formation (Supplemental S4C). Using a reporter gene construct containing NFκB-binding site, rCav-1 was able to elicit NFκB reporter activity in LNCaP WT cells or restore the decreased activity in Du145 shCav1 cells (Fig. 5b). Indeed, both phosphorylated RelA (p-p65) and phosphorylated NFκB1 (p-p50) levels were elevated in LNCaP Cav1OE cell compared with LNCaP Vc, in contrast, both RelA and NFκB1 were inactivated in Du145 shCav1 cells, (Fig. 5c). Also, NFκΒ inhibitor was able to inhibit the mRNA expression of Yamanaka factors in both LNCaP Cav1OE and Du145 Vc cells (Fig. 5d). As expected, NFκΒ inhibitor was able to block the inductive effect of rCav-1 on the expression of Yamanaka factors and NED-related factors in WT LNCaP cells (Fig. 5e). Analysis of the clinical data obtained from cBioPortal for Cancer Genomics (http://cbioportal.org) indicated a significant positive correlation of Cav-1 and NFκB1 mRNA correlation (Person correlation = 0.43; *p* value = 2.921 × 10^−6^) but less-significant correlation was found with RelA (Person correlation = 0.10; *p* value = 0.121) (Supplemental S4D) or non-canonical NFκB subunits (NFκB2, RelB; data not shown). Taken together, NFκΒ signaling pathway plays an important role in Cav-1-induced CSC or NED phenotypes.

### Cav-1 promotes cell migration and invasion of PCa via EMT

**Fig. 6:**
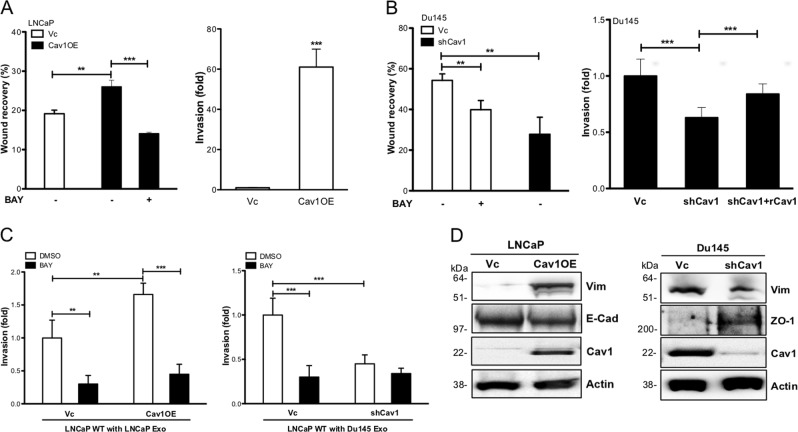
Cav-1-elicited NFκB signaling pathway promotes cell migration, invasion, and EMT. **a** The cell migratory or invasive abilities in LNCaP (Vc vs. Cav1OE) was measured using wound healing or Transwell invasion assay, respectively. **b** The cell migratory or invasive abilities in Du145 (Vc vs. shCav1) were measured using wound healing or Transwell invasion assay, respectively. **c** The effect of NFκB inhibitor (BAY, 5 µm) on cell invasion activities was measured in exosomes-treated WT LNCaP cell after 24h incubation. **d** The expression of EMT markers in LNCaP (Vc vs. Cav1OE) (Left panel) and Du145 (Vc vs. shCav1) (Right panel). All data were shown as mean ± SD (*n* > 10), *P < 0.5, **P < 0.01, ***P < 0.001

EMT is closely associated with CSC^43,44^ in which epithelial cell transdifferentiate into mesenchymal cell leading to accelerated cell migration and invasion. We noticed that LNCaP Cav1OE cells exhibited significantly higher cell migratory ability as well as invasive activities compared with LNCaP Vc cells, which could be blocked by NFκΒ inhibitor (Fig. 6a, Supplemental S5A). Similar results were found in Du145 shCav1 cells, with reduced cell migration and invasion when compared with Du145 Vc cells, and NFκΒ inhibitor could decrease both activities in Du145 Vc cells (Fig. 6b, Supplemental S5B). As expected, TDE-containing Cav-1 could increase the invasion of WT LNCaP cells, and NFκΒ inhibitor could diminish this activity (Fig. 6c).

For determining the effect of Cav-1 on EMT, we noticed that elevated Vimentin (mesenchymal marker) but reduced E-cadherin (epithelial marker) were found in LNCaP Cav1OE cells compared with control. In contrast, in Du145 shCav1 cells, Vimentin was decreased and zonula occludens-1 (tight junction protein in epithelia; Supplemental S5C) was increased compared with control (Fig. 6d). Moreover, ZEB1 and ZEB2 were characterized as EMT transcriptional factors can be regulated by the NFκB signaling pathway^45^; the elevation of both factors was detected in both Cav-1^high^ cells and NFκB inhibitor could suppress the expression (Supplemental S5D). To provide more evidence for the role of NFκB pathway in regulating EMT, the expression of Slug or Twist was elevated in Cav-1-positive cells but decreased in the presence of NFκB inhibitor (Supplemental S5E, F). Although no apparent morphology changes in LNCaP WT cells after treating with Exo-Vc or Exo-shCav1, Vimentin expression indeed increased in Exo-Vc treated cells (Supplemental S5G). These results indicate the critical role of Cav-1 in activating NFκB pathway leading to EMT that might contribute to cancer metastasis of PCa.

### Cav-1 delivered by TDE can promote radio- and chemo-resistance in PCa cell

**Fig. 7:**
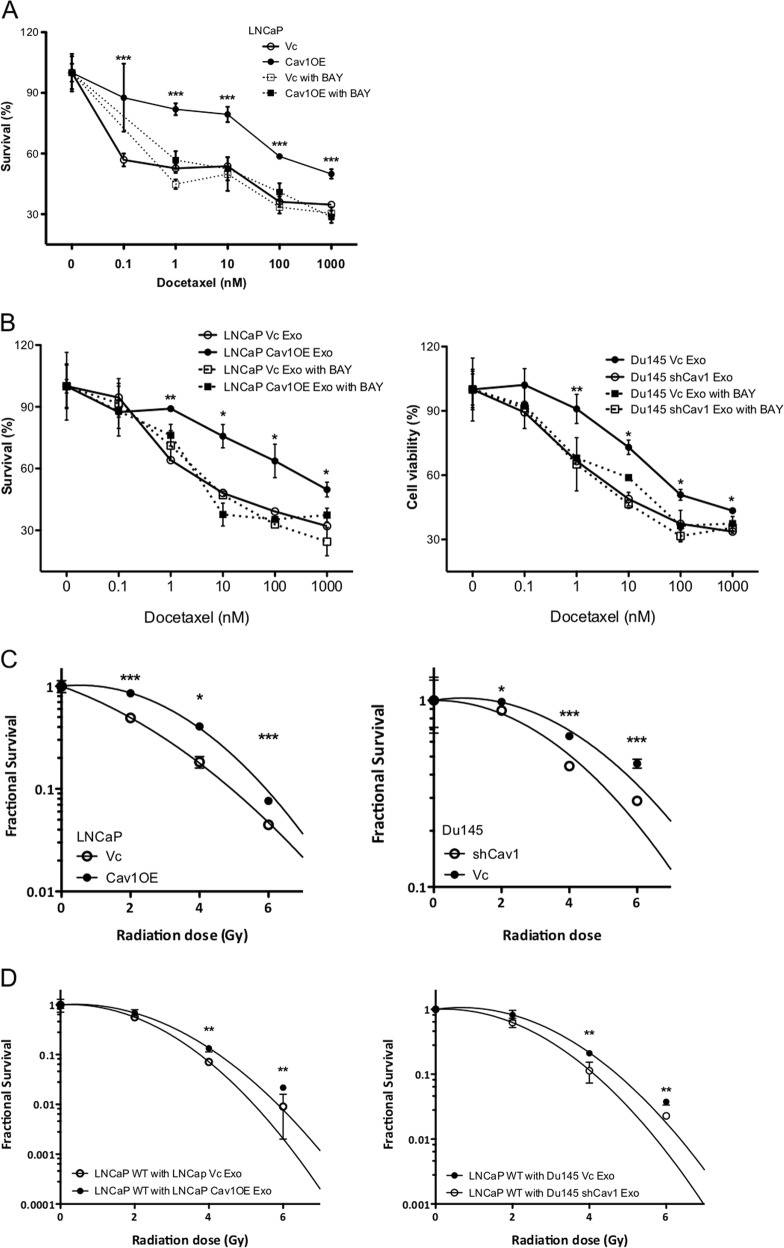
Cav-1 induces the resistance of PCa cells to chemotherapy or radiotherapy. **a** The effect of NFκB inhibitor (BAY, 5 µm) on the sensitivity of LNCaP (Vc vs. Cav1OE) cells exposed to different concentrations of Docetaxel was determined by MTT assay 48 h post treatment. **b** The effect of NFκB inhibitor (BAY, 5 µm) on the sensitivity of WT LNCaP cells incubated with exosomes from various sources and different concentrations of Docetaxel was determined by MTT assay 48 h post treatment. **c** The effect of radiation (0–6 Gy) on cell survival of LNCaP (Vc vs. Cav1OE) or Du145 (Vc vs. shCav1) was determined 14 days post radiation. **d** The effect of radiation (0–6 Gy) on cell survival of WT LNCaP treated with exosomes from various sources was determined 14 days post radiation. All data were shown as mean ± SD (*n* > 10), *P < 0.5, **P < 0.01, ***P < 0.001

CSC is often considered to be associated with chemo- and radio-resistance that lead to the failure of traditional therapies. We examined whether the increased Cav-1 could promote therapeutic resistance in PCa cells. Docetaxel is FDA-approved chemotherapy for CRPC patients, LNCaP Cav1OE cells acquired resistant to this agent compared with LNCaP Vc cells (IC_50:_ 3.97 vs. 7.18 nm) (Fig. 7a). Notably, in the presence of NFκB inhibitor, the Docetaxel resistance of LNCaP Cav1OE cells was diminished. In addition, TDE-containing Cav-1 could increase the Docetaxel resistance of cells but not TDE derived from Cav-1^low^ cells (Fig. 7b). As expected, the presence of NFκB inhibitor could reverse Docetaxel resistance from cells treated with TDE-containing Cav-1.

Similar to chemo-resistance data, both Cav-1^high^ cells showed higher cell survival under radiation treatment (from 0–6 Gy) (Fig. 7c). Furthermore, cells incubated with TDE-containing Cav-1 acquired radio-resistance compared with TDE derived from Cav-1^low^ cells (Fig. 7d). Taken together, these data indicate that an increased Cav-1 expression delivered by TDE to PCa cell is associated with therapeutic resistance phenotypes.

## Discussion

PCa is one of the leading causes of cancer-related death of males in United States. Despite many effective therapies for the primary disease such as surgery, radiotherapy, and hormonal therapy, many patients still experience recurrent disease. mCRPC representing the end-stage of this disease remains an incurable disease. The introduction of novel anti-androgens such as abiraterone acetate and enzalutamide has only slightly increased the survival of mCRPC patients^46^. Thus, identifying the molecular mechanisms as well as cell of origin associated with mCRPC will be critical for developing effective therapeutic strategies for this disease.

Recent studies indicate that CSCs may be the primary source leading to development, invasion, metastases, recurrence, and drug resistance of cancers^47,48^. Although classical stem cell model indicates that only a small subset of cells are capable of making progeny cells, CSC theory predicts that the genetically unstable carcinoma cells can rapidly become stem cells after the onset of de-differentiation or transdifferentiation program induced by either intrinsic or extrinsic signals^11^. In mCRPC, increased expressions of stem cell drivers or EMT markers have been identified from clinical specimens as well as animal models^14,49^, suggesting that CSC promotes mCRPC progression.

Cav-1 was originally identified as a structural protein of caveolae in lipid rafts^50,51^, which is a plasma membrane domain that regulates a variety of signaling pathways involved in cell growth and migration^42^. Although Cav-1 has been shown to play different roles in each specific cancer types, such as low expression of Cav-1 in prostate stroma contributes to tumor progression^52^, recent study have found elevated Cav-1 is associated with human PCa and genetically modified mouse model^24,53^. Our previous study and others have demonstrated the gain of function of Cav-1 in tumor growth^16,54^, particularly, Cav-1 expressed in PCa cells is secreted in an endocrine manner^55^. Consistently, elevated Cav-1 can be detected in the serum of CRPC patients^56,57^. It appears that elevated Cav-1 is found in androgen receptor (AR)-negative cells^58^. Noticeably, our data unveiled a significant elevation of Cav-1 expression in an AR-positive adenocarcinoma cell model containing both p53 and Rb gene knockout (both mutations are commonly found in CRPC patients associated with NED phenotypes)^30,31,59^; this cell line acquires lineage plasticity owing Sox2 gene expression through transdifferentiation. Also, in the gene expression analysis have shown that p53 and Rb1 double knockout altered the gene set that relates to stem cell and epigenetic reprogramming, suggesting that the CSC population might be the source of NEPC cell^60^. Thus, ectopic expression of Cav-1 in an androgen-responsive PCa cell can increase the prostate sphere formation as well as the expression of Yamanaka factors (four transcriptional factors required for inducing pluripotent stem cell). Knocking down endogenous Cav-1 expression in a CRPC cell significantly reduces the prostate sphere formation, the expression of Yamanaka factors, and in vivo tumorigenicity. Apparently, Cav1OE PCa cells are resistant to either chemotherapy or radiotherapy. Altogether, these data support the critical role of Cav-1 in CSC development and underlying the onset of mCRPC associated with therapy resistance.

Several signaling pathways have been linked to promote CSC phenotype, including Wnt/β-catenin, Sonic Hedgehog, and Notch signaling pathways^61^. We describe canonical NFκB signaling cascade as the key pathway in CSC development. This finding is consistent with previous studies that NFκB is upregulated in tumor initiating population purified from human PCa xenograft tumors^62^ and a good clinical correlation of NFκB signaling pathway with PCa progression^63,64^. Notably, we found that NFκB inhibitor treatment could also block Cav-1 expression (Supplemental S6). Studies also proposed therapy strategies that target NFκB signaling pathway^65,66^, which strengthen our conclusion that Cav-1-NFκB signaling pathway involved in PCa progression. Mechanistically, Cav-1 has been shown to associate with IKKβ leading to NFkB activation in liver cancer cells^67^. Thus, whether similar mechanism is involved in prostate CSC formation required further investigation.

Exosomes are small extracellular vesicles with a double membrane structure that originate from intracellular multivesicular bodies, which carry different biomolecules including RNAs, proteins, and lipids and deliver these cargoes in an endocrine or paracrine manner. Knowing its native membrane structure and specific cargo payload, exosomes has been developed as a drug delivery system^68,69^. TDEs‐mediated intercellular communication is known to remodel tumor microenvironments and form premetastatic niches during cancer development^70^. In PCa, some evidence indicates that exosomes produced by tumor surrounding microenvironment can promote cancer progression^71,72^, however, the role of TDEs in PCa development is not well studied. Herein, we identified Alix and CD9 as the common biomarkers for PCa TDEs and demonstrated that the presence of Cav-1 in TDEs from PCa functioned as a potent inducer of CSC in recipient cells by triggering the same signaling pathway as membrane form of Cav-1, implying TDEs can be a potential prognostic marker for CRPC patients. In line with our result, other studies did use Alix and CD9 as exosome markers in patient urine^73^, and plasma samples^74^. Although previous studies have found high level of Cav-1 from the serum of PCa patients compared with benign prostatic hyperplasia patients^56,57^, further studies are needed to validate the applicability of TDEs in CRPC prognosis. Overall, this study highlights a novel functional role of Cav-1 in CRPC progression associated with CSC-NED transdifferentiation, which identifies Cav-1 and its downstream pathway as potential therapeutic targets. In addition, the presence of Cav-1 in PCa TDEs offers a potential prognostic marker for CRPC patients from liquid biopsy.

## Materials and methods

### Cell culture

LNCaP, Du145 were obtained from American Type Culture Collection and were maintained in RPM1640 (Sigma-Aldrich, St. Louis, MO). Stable Cav-1 overexpressing (Cav1OE) and control (Vc) were generated from LNCaP cell as previously described^16^. CRISPR-Cas9 Rb/TP53 knockdown (Rb^-^/p53^-^) and its control (Con) were kindly provided by Dr. Ping Mu^31^. Stable Cav-1 knockdown (shCav1) and control (Vc) were generated by using pLKO.1-shCav1 (shCav1#1: TRCN0000007999; #2: TRCN0000008000; #3: TRCN0000008002) (RNAi core, Academia Sinica, Taipei, Taiwan). All culture media were supplemented with 10% fetal bovine serum (Life Technologies, Carlsbad, CA), and 100 U/ml penicillin with 100 μg/mL streptomycin (Life Technologies) and cells were maintained at 37 °C with 5% CO_2_ in a humidified incubator. For CRISPR-Cas9 Rb^−^p53^−^ and control cells, RPMI medium was supplemented with additional 1% HEPES (4-(2-hydroxyethyl)-1-piperazineethanesulfonic acid; Gibco, Invitrogen, Carlsbad CA USA), 1% GlutaMax (Gibco), 1% Sodium Pyruvate (Gibco). Each cell line was authenticated with the short tandem repeat profiling by Genomic Core in UT Southwestern periodically. MycoAlert kit (Lonza Walkersville, Inc. Walkersville, MD) was used to confirm mycoplasma free condition every month.

### Quantitative real-time PCR (qRT-PCR)

Total RNA was extracted using Maxwell 16 LEV SimplyRNA Purification Kit (Promega, Madison, WI) and 1 μg RNA was reversely transcribed into cDNA using iScript cDNA Synthesis Kit (BioRad, Hercules, CA). Real-time PCR analysis was set up with SsoAdvenced Universal SYBR Green Supermix Kit (BioRad) and carried out in MyiQ thermal cycler (BioRad). The relative level of target mRNA was determined by normalizing 18S rRNA. All experiments were repeated at least three times in triplicates each time. Primer sequences were described in Supplemental Table 2.

### Prostate sphere assay

The prostate sphere assay was based on previous reports^14,75^. Cells (100 cells) were seeded in 96-well ultralow attachment plates (Corning) in 200 µl of sphere culture medium (serum free culture medium supplemented with 2% B27 (Gibco), 20 ng/ml EGF (Gibico) and 20 ng/ml FGF-2 (Gibico) for 14 days. During the incubation, 20 µl of sphere culture medium was replenished every 3–4 days. Number of Spheres was counted at 14 days after plating.

### Flow cytometry

For determining stem cell population, cells were incubated with allophycocyanin-conjugated human monoclonal CD24 antibody (Ab) (BD Biosciences, San Jose, CA) and phycoerythrin-conjugated human monoclonal CD44 Ab (BD Biosciences) for 30 min then subjected to cytometry analysis (FACS Calibur, BD Biosciences).

### Invasion assay

Invasion assay was performed using 8.0 μm polycarbonate membrane Transwell (Corning Costar, Corning, NY) coated with 100 µl of 2% Matrigel (Corning). Cells (1 × 10^5^ cells in 100 µL) were plated in the upper chamber that was inserted into the lower chamber filled with 500 µL of 10% fetal bovine serum (FBS) medium. After 8 h of incubation, cells in the apical side of upper chamber were removed and transmigrated cells on the basal side of upper chamber were fixed in 4% paraformaldehyde for 10 min. Fixed cells were stained with 1% Crystal Violet to determine cell number.

### Wound healing assay

Cells were seeded onto 12-well plates and incubated for 16–18 h for cell attachment. Wound scratches were made by 200 µl tip and photographed for the width of scratch after removing cell debris with PBS then cells were incubated with 5% FBS medium for 48 h. Subsequently, photograph was taken every 24 h and cell migratory ability was calculated the percentage of recovery rate. The experiments were performed in triplicate independently.

### Transfection and luciferase reporter assay

In all, 70% confluence cells (5 × 10^4^ cell/well) were seeded onto 24-well plate 16–18 h before transfection. Cells were co-transfected with luciferase reporter plasmids (0.5 µg/well) and Renilla luciferase-expressing internal control, pRL-TK (Promega; 2 ng/well). Transfections were performed using JetPEI transfection kit (Q-Bio Gene, Irvine, CA, USA) according to the manufacturer's instructions. In all, 48h after transfection, the wells were rinsed with PBS, and cells were harvested with 200 µl of passive lysis buffer (Promega). Following incubation on ice for at least 30 min, the insoluble debris was removed by centrifugation at 4 °C for 2 min at 13,000 rpm. In all, 20 µl of the supernatant were processed for sequential quantitation of both firefly and Renilla luciferase activity (Dual-Luciferase Assay System, Promega) using a Monolight TD luminometer (Turner Designs). The activity of the Renilla reporter plasmid was used for normalization of transfection efficiency. All transfection experiments were performed in triplicates independently.

### Western blot and slot blot analyses

Cells were rinsed with PBS and lysed in ice-cold lysis buffer (150 mmol/L NaCl, 1% Triton X-100, 0.5% sodium deoxycholate, 0.1% SDS, 50 mmol/L Tri (pH 8.0), protease inhibitor cocktail (Roche)), and cell debris was removed by centrifugation at 4 °C for 10 min at 13,000 rpm. For western blot, equal amount of protein was subjected to electrophoresis on NuPAGE gels (Life Technologies) then transferred onto nitrocellulose membranes. For slot blot, equal amount of protein samples and standard were spotted onto the nitrocellulose membranes by using Milliblot S immunodot sets (Millipore Corp., Bedford, MA). Subsequently, membranes were incubated with 2% non-fat dry milk (w/v) for 1 h and then washed in PBS containing 0.1% Tween 20 then incubated with primary Abs (Supplemental Table 1). Appropriate secondary antibodies conjugated with horseradish peroxidase and enhanced chemiluminescence were used to detected target proteins.

### In vivo tumorigenicity assay

All animal work was approved by the Institutional Animal Care and Use Committee. Serial dilutions of Du145 cells were subcutaneously injected into 6–8-weeks-old male SCID mice. In addition, Du145 shCav1 (10,000 cell/site) were mixed with Matrigel (1:1 ratio, final volume = 100 μl) and subcutaneously injected in SCID mice. TDEs (total protein = 40 μg/site 80–120 ng Cav-1) were treated twice-a-week for 4 weeks^76^. The tumor volume was determined by caliper and calculated using the ellipsoid formula (π/6 × length × width × depth).

### Preparation and characterization of exosome from CM

Cells (1 × 10^6^) were grown in p-150 culture plate for 3~5 days until reaching 90% confluence using 10% exosome-free FBS medium then culture media from 3–5 plates were collected for exosome purification using gradient centrifugation (500 × *g* for 10 min, 3,000 × *g* for 20 min). Supernatant was passed through 0.22 µm filters as CM. Subsequently, CM was centrifuged at 100,000 × *g* for overnight and washed once with PBS, then centrifuged at 100,000 × *g* for 2 h to collect the pellet for exosome that was re-suspended in 100 μl PBS and stored at − 80 °C until use.

Exosome size was determined by Zetasizer (Malvern Zetasizer Nano-ZS, Malvern Instruments, Worcestershire, UK) and the particle number was measured by using EXOCET Exosome Quantitation Assay kit (SBI, System Biosciences, CA) according to the manufactures’ protocol.

### Radiation and clonogenic survival assay

Cells were irradiated in ambient air using a JL Shepherd Mark 1–68 137Cs irradiator at a dose rate of 3.47 Gy/min. A serial dilution of cell suspension was plated onto 60-mm dish, after cell attachment, cells were exposed to the increasing doses of IR (0, 2, 4, 6, and 8 Gy). After 6 days of incubation, the colonies were fixed and stained with 4% formaldehyde in PBS containing 0.05% crystal violet. Colonies containing > 50 cells were counted. Surviving fraction was calculated as (mean colony counts)/[(cells inoculated for irradiation) × (plating efficiency)], in which plating efficiency was defined as (mean colony counts)/(cells inoculated for control). The data are presented as the mean ± SEM and the curve *S* = *e*–(αD+βD2) was fitted to the experimental data using GraphPad Prism software.

### Bioinformatics and statistical analyses

PCa patient data from The Cancer Genome Atlas database were archived in cbioportal for Cancer Genomics (http://www.cbioportal.org)^77,78^. MSKCC data set with mRNA Expression z scores vs. normal (z scores threshold ± 2) was used for gene correlation analysis^79^. In brief, MSKCC Prostate Oncogenome Project contains 181 primary, 37 metastatic PCa samples, 12 PCa cell lines, and xenografts.

All experiments were performed for at least three repeats. All numerical data represent mean and standard deviation. Student *t* test was used for the determination of statistical significance between groups (*p* < 0.05). All statistical analyses were performed with GraphPad Prism software.

## Supplementary information

Supplemental Materials
