# Supplementary material for: The paracrine induction of prostate cancer progression by caveolin-1
Source: Cell Death Dis. 2019 Nov 4;10(11):834. doi: 10.1038/s41419-019-2066-3 (PMC6828728; doi:10.1038/s41419-019-2066-3)

Supplementary Information-Text summary

Table:
S1 Antibodies used in study
S2 Primer pairs used in study

Figure S1 The expression of Cav-1 and CSC biomarkers in PCa cell lines.
(A) The expression profile of Cav-1 protein in different PCa cell lines. (B) Elevated Cav-1 protein expression in LNCaP Cav1OE cells compared with LNCaP Vc cells. (C) The effect of three specific Cav-1 shRNA on the expression of Cav-1 in Du145 Vc. (D) The expression of CSC biomarkers (CD24 or CD44) mRNA expression in LNCaP and Du145 cells. (E) The expression of ALDH1A1 in LNCaP and Du145 cells. Data were shown as mean ± SD from three independent experiments (n = 10).

Figure S2 The expression of CSC and NED factors in PCa cell lines.
(A) The expression of p53, Rb1 and Cav1 in LNCaP Control, Rb1 knockdown (KD), P53 knockdown, and double knockdown ($RB^-/p53^-$) cells. (B) The expression of Yamanaka factors in RB and p53 knockdown cells. (C) The expression of ALDHA1 in Rb-/p53- cells. (D) The expressions of BRN2 (Left panel) and CGA (Right panel) gene were determined in LNCaP (Cav1OE or Vc) and Du145 (Vc shCav1) cells. Data were shown as mean ± SD from three independent experiments (n = 10).

Figure S3 Characterization of Cav-1 levels in CM from TDE fraction and TDE-free fraction.
Standard curve of Cav-1 protein measurement. (B) Cav-1 expression was detected in LNCaP and Du145 derived exosomes and exosome depleted CM (Exo(-)CM) by slot blot. Alix and CD9 were used the internal TDE markers. Same amount of total protein from each sample was loaded. (C) Prostate sphere formation was determined using WT LNCaP incubated with exosome-free culture medium or Exo(-)CM.

Figure S4 The target validation and growth effect of a panel of small molecule inhibitors.
(A) The effect of individual small molecule inhibitor on specific signaling effector. PI3K/Akt (LY294002; 1 μM), Erk (PD98059; 2 μM), p38 (SB202190; 100 nM), JNK (SP600125; 100 nM), Wnt (IWP2; 30 nM), NFκB (BAY11-7082; 5 μM), and Gli1/2 (GANT; 5 μM). (B) The effect of small molecule inhibitors on in vitro growth of LNCaP Cav1OE and Du145 Vc was determined at 24h and 48h after treatment by MTT assay. (C) The efficiency of p65 and p50 siRNA single knockdown in Du145 cell (Left), and their sphere formation activity (Right). (D) The correlation of canonical NFκB subunit (NFκB1 and RelA) was analyzed by MSKCC with mRNA Expression z-Scores vs. normal (z-Scores threshold ± 2) dataset obtained from Cbioprotal.

Figure S5 The role of Cav-1-elicited NFκB signaling pathway in promoting cell migration, cell invasion and EMT driver gene expression.
(A) The effect of Cav-1 or NFκB inhibitor (BAY, 5 μM) on cell migration or invasion of LNCaP Vc or Cav1OE cells. (B) The effect of Cav-1 or NFκB inhibitor on cell migration or invasion of Du145 Vc or shCav1 cells. (C) ZO-1 expression in Du145 cells. Bar = 10μm. The

effect of NFκB inhibitor on ZEB1 or ZEB2 gene expression in LNCaP (Vc or Cav1OE) or Du145 shCav1 cells with or without rCav-1. (D) The effect of NFκB inhibitor on Slug mRNA expression. (E) The effect of NFκB inhibitor on Twist protein expression. (F) Vimentin staining in exosome treated LNCaP WT cell. Bar = 20μm.

Figure S6 Cav1 expression was altered by NFκB inhibitor.
The expression of Cav1 in Du145 after NFκB inhibitor (BAY 5μM) treatment. Data was shown as mean ± SD from three independent experiments (n = 10).

Supplementary Figure

Supplemental Figure S1

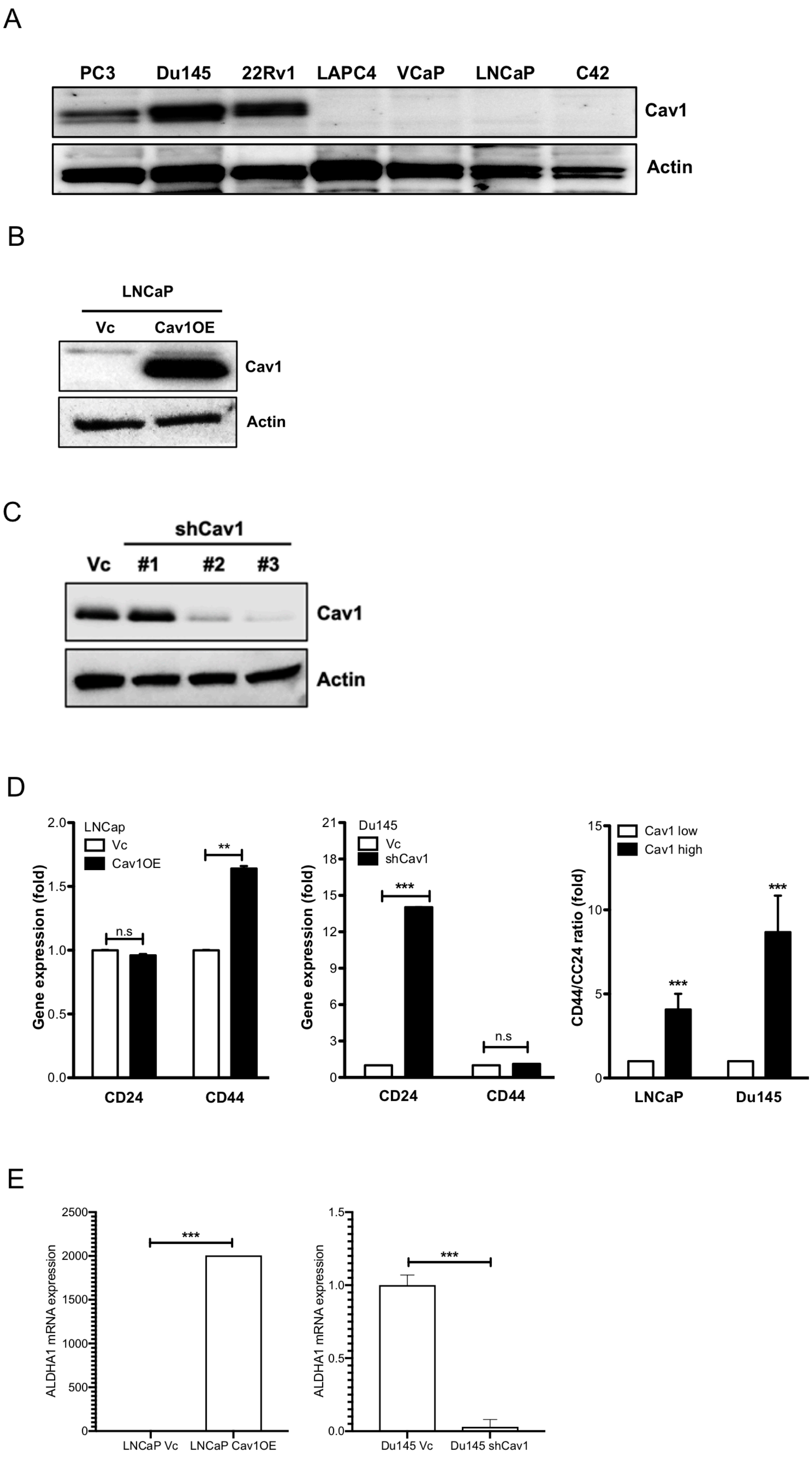

Supplemental Figure S2

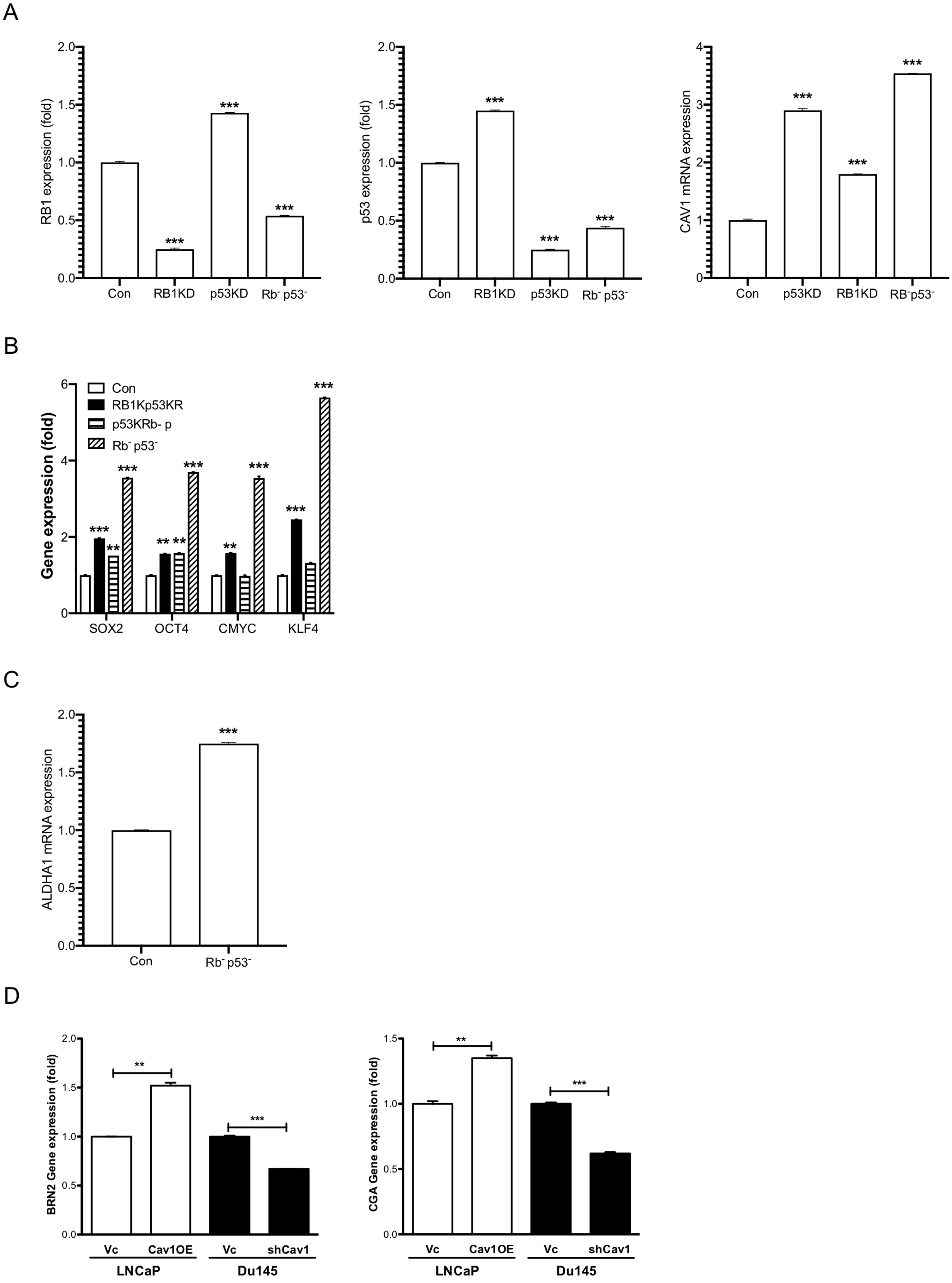

Supplemental Figure S3

A

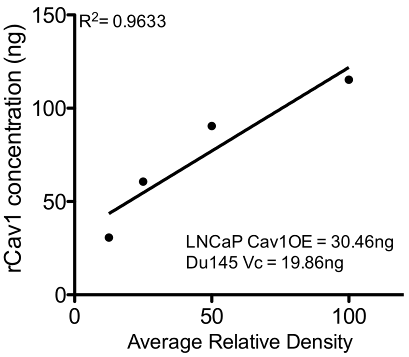

B

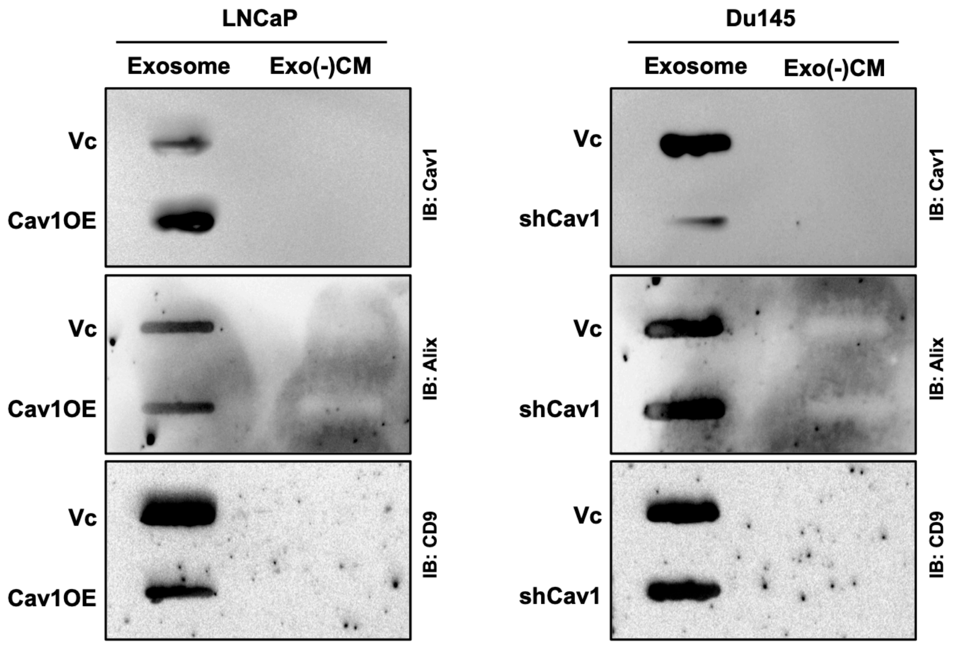

C

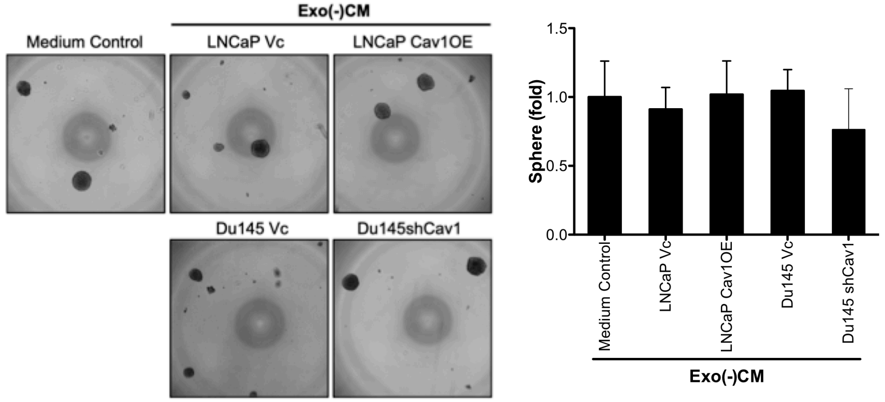

Supplemental Figure S4

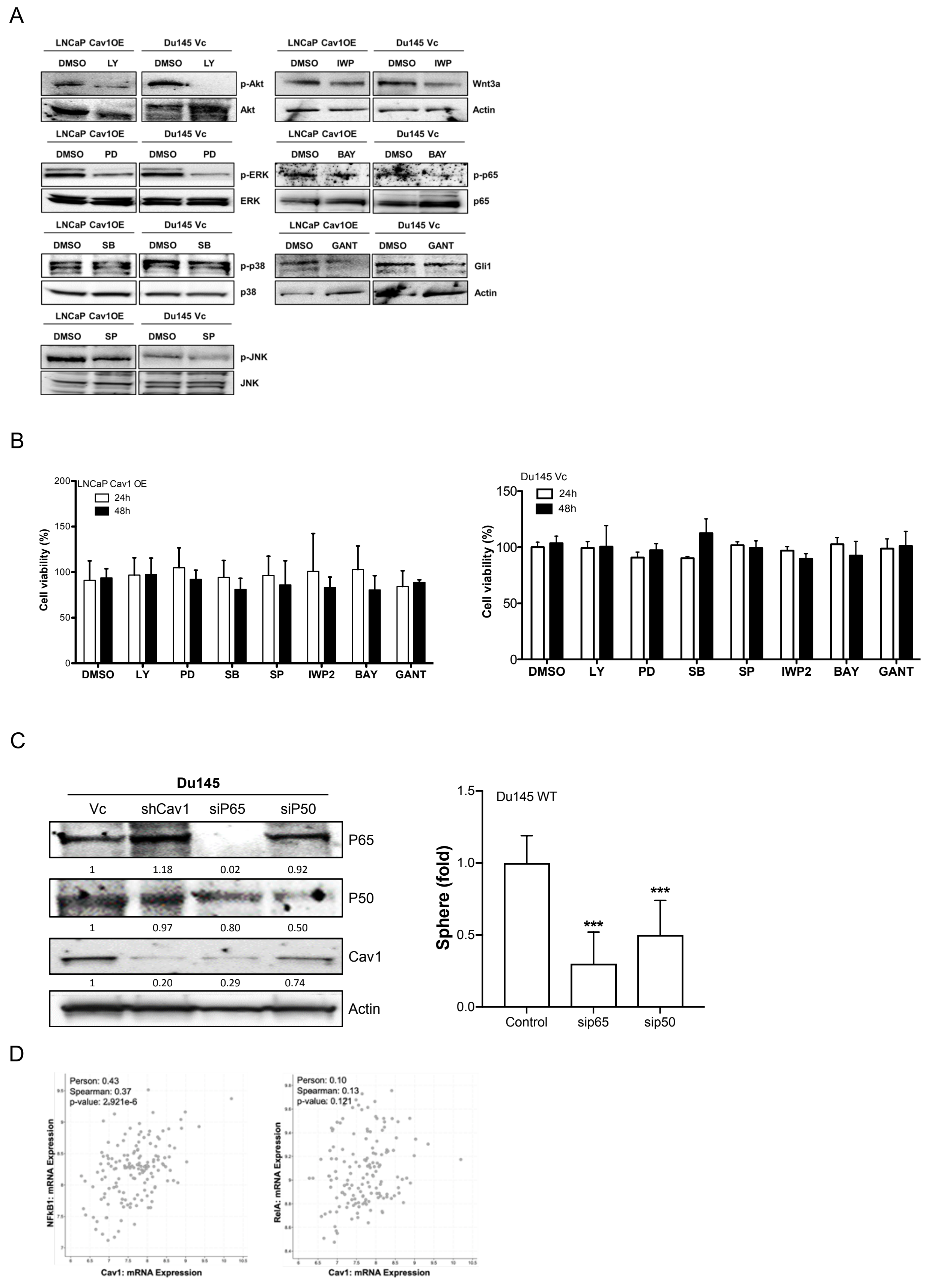

Supplemental Figure S5

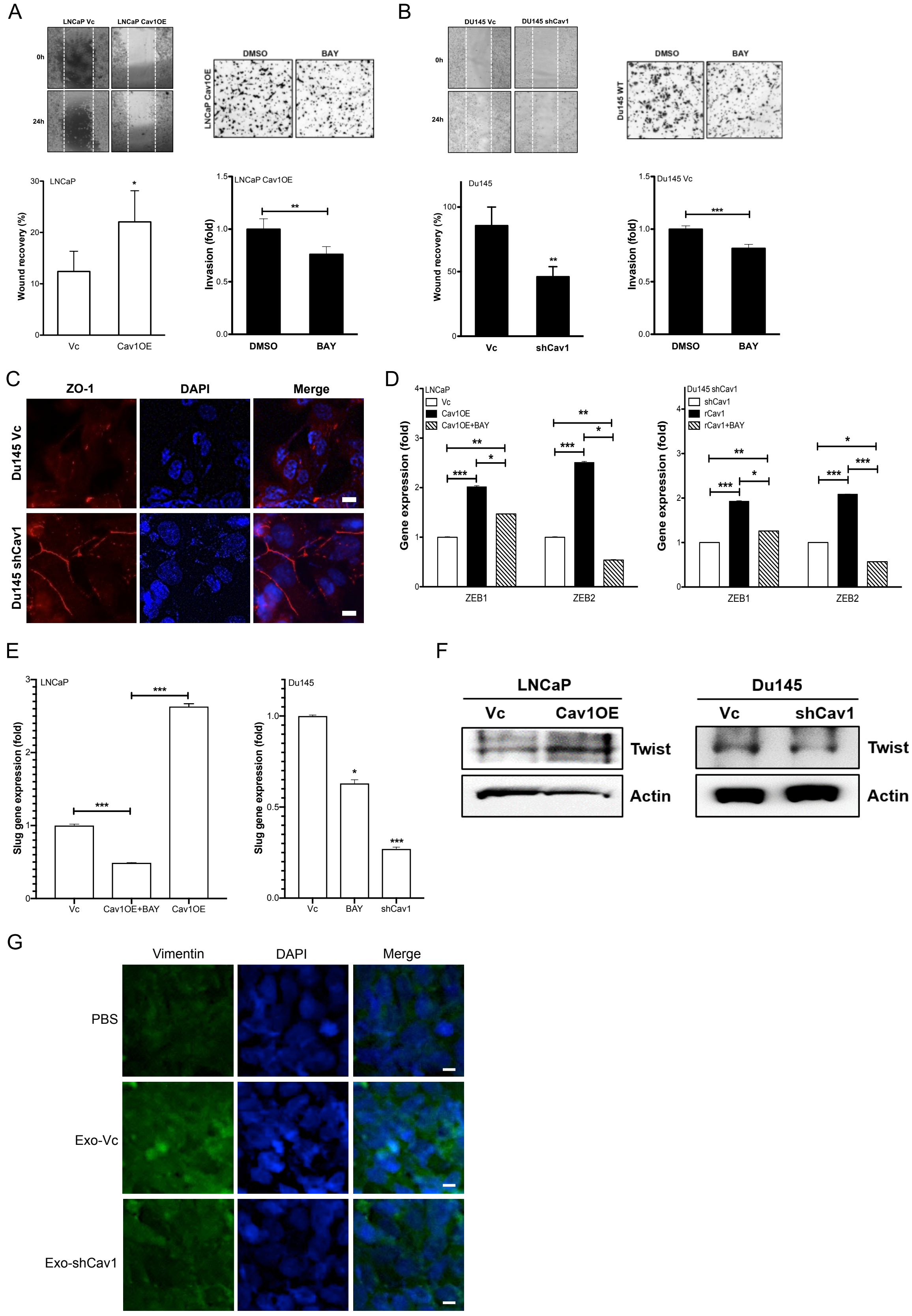

Supplemental Figure S6

A

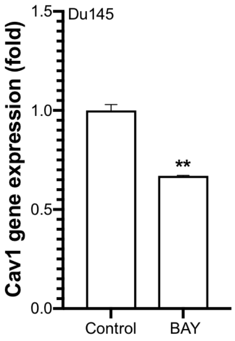

Supplement: Supplementary file 1 — Supplemental Materials [file 41419_2019_2066_MOESM1_ESM.pdf]
